# A Bibliographic Dataset of Health Artificial Intelligence Research

**DOI:** 10.34133/hds.0125

**Published:** 2024-04-05

**Authors:** Xuanyu Shi, Daoxin Yin, Yongmei Bai, Wenjing Zhao, Xin Guo, Huage Sun, Dongliang Cui, Jian Du

**Affiliations:** ^1^ Institute of Medical Technology, Peking University, Beijing, China.; ^2^ National Institute of Health Data Science for Huage Sun, Peking University, Beijing, China.; ^3^ Advanced Institute of Information Technology, Peking University, Hangzhou, China.; ^4^ Department of Cardiology and Institute of Vascular Medicine, Peking University Third Hospital, Beijing, China.; ^5^ Institute of Health Informatics, University College London, London, UK.; ^6^ University of Science and Technology Beijing, Beijing, China.

## Abstract

**Objective:** The aim of this study is to construct a curated bibliographic dataset for a landscape analysis on Health Artificial Intelligence (HAI) research.

**Data Source:** We integrated HAI-related bibliographic records, including publications, open research datasets, patents, research grants, and clinical trials from Medline and Dimensions.

**Methods:** Searching: Relevant documents were identified using Medical Subject Headings (MeSH) and Field of Research (FoR) indexed by 2 bibliographic databases, Medline and Dimensions. Extracting: MeSH terms annotated from the aforementioned bibliographic databases served as the primary information for our processing. For document records lacking MeSH terms, we re-extracted them using the Medical Text Indexer (MTI). Mapping: In order to enhance interoperability, HAI multi-documents were organized using a mapping system incorporating MeSH, FoR, The International Classification of Diseases (ICD-10), and Systematized Nomenclature of Medicine Clinical Terms (SNOMED CT). Integrating: All documents were curated based on a pre-defined ontology of health problems and AI technologies from the MeSH hierarchy.

**Results:** We collected 96,332 HAI documents (publications: 75,820, open research datasets: 638, patents: 11,226, grants: 6,113, and clinical trials: 2,535) during 2009 to 2021. On average, 75.12% of the documents were tagged with at least one label related to either health problems or AI technologies (with 92.9% of publications tagged).

**Summary:** This study presents a comprehensive pipeline for processing and curating HAI bibliographic documents following the FAIR (Findable, Accessible, Interoperable, Reusable) standard, offering a valuable multidimensional collection for the community. This dataset serves as a crucial resource for horizontally scanning the funding, research, clinical assessments, and innovations within the HAI field.

## Introduction

For a long time, people have been facing serious health problems such as the aging of the population, chronic disease burdens, outbursts of infectious diseases, environmental changes, etc. Artificial intelligence (AI) is a novel technology used to solve challenging real-world problems autonomously that traditional technologies find difficult to solve [1]. In medicine, AI has been used to utilize existing medical data to discover the potential relationships and patterns that can inform clinical diagnosis, treatment, and outcomes evaluation [2].

The development and application of AI have attracted widespread attention from academia and industry. Stanford University began to publish annual AI Index Reports in 2017 [3]. By collecting, analyzing, and tracking AI developments, the report provided synthesized data to keep decision-makers updated on the leverage of the most advanced AI technologies in the spheres of economy, education, and so on. Additionally, researchers published a qualitative review of recent AI advances in health [4], summarizing the excellence of AI in various medical specialties and discussing the current challenges. Another review focuses on the major advances of AI in medical image analysis, summarizing the current progress, challenges, and opportunities [5]. Nevertheless, the aforementioned papers either provided an overarching picture or were based on experts’ selection of AI applications in specific areas of medicine that are published in high-ranking peer-reviewed medical journals.

The evolution of AI technology is rapid, and its applications in various medical and health disciplines are diverse. To effectively analyze the impacts and challenges of AI in medicine, a deeper understanding of specific domain knowledge and insights from practical experience are crucial. This involves recognizing the unique developmental trajectories of AI within different medical specialties. For instance, the way AI is integrated into radiology, with its focus on image analysis and interpretation, differs greatly from its application in areas like patient care and hospital management; AI's role lies in streamlining operations and enhancing patient experience, which requires a different set of considerations. That is why simply providing a generalized, inclusive summary report of AI and its application in health and medicine falls short of adequately reflecting the unique advancements and specific challenges faced by each health discipline. Such an approach overlooks the distinct ways AI technology is being integrated and utilized in various medical fields, from diagnostics to treatment planning and from patient care to medical research. Each discipline has its own set of complexities, goals, and benchmarks for success, which necessitate a more nuanced and detailed analysis. This is further supported by findings from a survey interview, where researchers expressed a preference for analyzing the underlying data by themselves rather than simply being given synthesized reports [6]. However, there is no comprehensive and regularly updated bibliographic dataset for researchers to navigate according to their specific needs and develop their understanding of the updates and gaps in their fields.

In this study, we introduced a curated dataset that includes health-related publications, open-source datasets, patents, grants, and clinical trials with applications of AI from 2009 to 2021. We not only retrieved papers from scientific journals but also included documents from preprint websites, clinical trial platforms, and other types of data from Dimensions [7]. Our objective in providing this dataset is to offer a comprehensive resource for researchers, policymakers, and investors, enabling them to probe deeply into the unique challenges and opportunities within their specific areas of health and medicine. By facilitating access to detailed and relevant data, we aim to equip these stakeholders with the necessary tools to develop a thorough understanding of the current landscape in their fields. This in-depth insight is crucial for identifying gaps, trends, and potential areas of growth. By bridging the gap between data availability and actionable knowledge, we hope to foster informed decision-making, encourage evidence-based policies, and spur investments in areas that are pivotal for advancing healthcare and medical research.

## Methods

In this study, we proposed a quantitative method of acquiring and curating the Health Artificial Intelligence (HAI) dataset. In order to accomplish the goal of making the dataset informative, indexable, and applicable, there were 3 main steps involved to construct the HAI dataset. Firstly, we utilized PubMed [8] and Dimensions [7] as the data sources and define HAI documents with the adoption of the different bibliographic databases. Secondly, more detailed categories were extracted using a medical natural language processing tool developed by the National Library of Medicine (NLM) and then mapped to other vocabularies Systematized Nomenclature of Medicine Clinical Terms (SNOMED CT) and International Classification of Diseases (ICD-10). We also labeled the documents with specific health problems and AI technologies using the acquired sub-categories. Lastly, we presented a well-curated dataset with statistics of annotation. Visualizations of flowing and interactions between health and AI in this dataset are presented in Results.

**Fig. 1.**
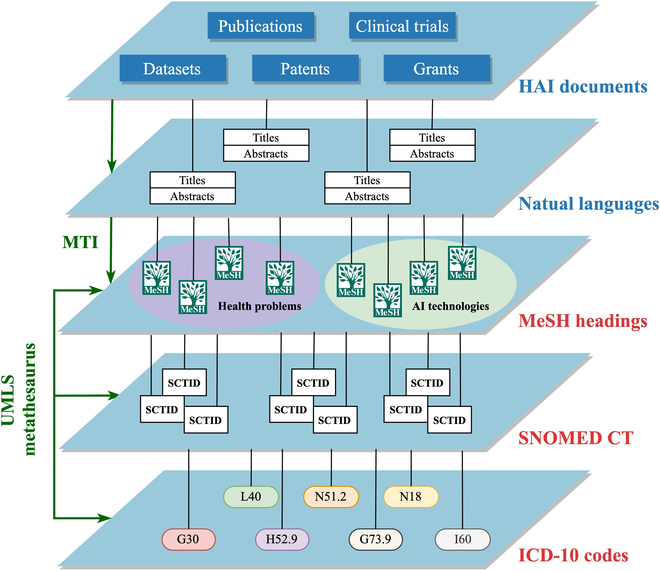
A hierarchical overview of the study pipeline.

Figure 1 illustrates the overall design of this study. From the top of the hierarchy, we acquired titles and abstracts from 5 types of HAI documents. Next, by taking advantage of the MTI tool, we extracted Medical Subject Headings (MeSH) terms from the acquired natural languages and then combined the terms into health problems and AI technologies. Lastly, under the UMLS (Unified Medical Language System) metathesaurus system, we mapped the MeSH terms into additional standard medical vocabularies including SNOMED CT and ICD-10 for more disease-oriented classifications.

Briefly speaking, we aim to build a pipeline that identifies standard vocabularies and classifications from natural languages in HAI documents and regroup them into HAI categories, for the ultimate purpose of providing researchers the ability to retrieve and analyze the past trends and latest hotspots quantitatively in HAI.

### Data searching

**Fig. 2.**
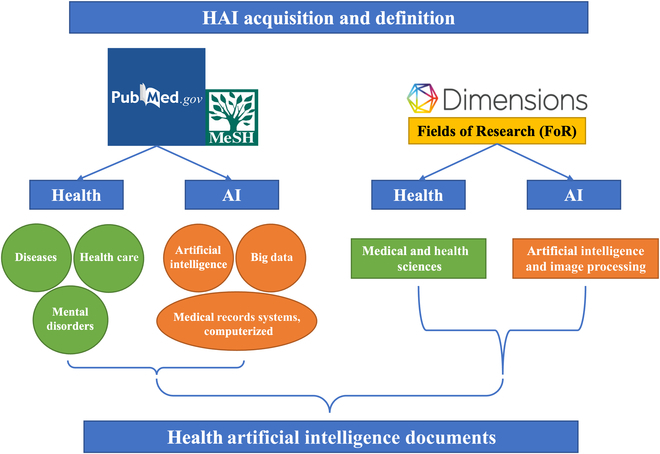
The acquisition strategy for HAI documents.

The work of data collection on the Web needs to be carried out with great care [9]. Inappropriate data collection methods lead to false and unscientific results. In this study, we used the intersection of documents in health and AI to precisely define and acquire the HAI dataset. Figure 2 shows the data acquisition strategy for defining HAI documents. Firstly, we took advantage of MeSH terms to index HAI documents from PubMed. Meanwhile, PubMed does not include all types of documents such as conference papers, patents, funding, etc. Secondly, we acquired HAI documents from Dimensions using FoR to complete the dataset. The full dataset is composed of 5 types of documents: publications, datasets, patents, grants, and clinical trials. While publications were acquired from both PubMed and Dimensions, the other documents were acquired from Dimensions only, which is a large bibliographic platform that integrates multiple sources of documents. The detailed retrieval and acquisition strategy is explained in the following subsections.

#### Publications

We acquired HAI publications from 2 bibliographic databases: Medline and Dimensions. Medline, the NLM biomedical literature database, contains over 28 million references. Medline records can be indexed using MeSH terms. In this study, we proposed an indexing method using MeSH terms for defining HAI publications. Generally, in Medline, each article is labeled with multiple MeSH terms, with some of them being major MeSH terms for accurately describing publication topics.

Specifically, for “Health”, we used categories “Diseases”, “Mental Disorder”, or “Health Care” and all their sub-categories. For “Artificial Intelligence”, we used “Artificial Intelligence”, “Big Data”, or "Medical Records Systems, Computerized” and all sub-categories. Following this procedure, we retained the intersection of publications from both types, defining it as the first part of our HAI publications.

We acquired the other part of the dataset from Dimensions, using the classification system to define HAI publications. We selected publications with FoR “11 Medical and Health Sciences” as the health category and “0801 Artificial Intelligence and Image Processing” as the AI category, then intersected and merged the results from Medline with our publications dataset. After limiting the time window to 2009 to 2021, we obtained 75,820 HAI publications from Medline and Dimensions, which we defined as HAI publications.

#### Open research datasets

The FAIR principle was introduced in 2016 to make research data “Findable, Accessible, Interoperable, and Reusable” [10]. Adhering to the FAIR principle in research data not only enhances the visibility of research but most importantly increases the reuse of research data, thereby enabling validations and improvements in further research. In scientific research, resource sharing plays a vital role to achieve efficiency, power, and rigor [11]. It is believed that reusing and reorganizing already-generated data would be beneficial in case of expensive and duplicated research [12]. High-quality data sources can help AI succeed in clinical classification and prediction tasks [13]. In the development of AI algorithms and reviewing publications in health care, researchers often need to track the original dataset to verify the efficacy and efficiency of the proposed methods. FigShare [12] stores and shares user-uploaded data including figures, datasets, software, etc., from manuscripts, preprints, and publications. This database covers most of the academic categories including “Health Sciences” and “Information and Computing Sciences”, which this study finds interesting. Dimensions links FigShare datasets with publications, providing a massive, linked, and structured collection of shared scientific resources. In this study, we used Dimensions to acquire HAI datasets with the FoR system (intersection of “11 Medical and Health Sciences” and “0801 Artificial Intelligence and Image Processing”), resulting 638 indexed datasets acquired.

#### Patents

According to the WIPO (World Intellectual Property Organization) AI report in 2019, 12% of all AI-related patents are filed in life and medical sciences [14]. This significant proportion of HAI patents highlights the importance of examining metadata and conducting further curation of patents. For HAI patents, we used FoR to define the dataset and obtain data from Dimensions. We employed the same FoR strategy for HAI publications, intersecting “11 Medical and Health Sciences” and “0801 Artificial Intelligence and Image Processing”. We identified 11,226 HAI patents from 2009 to 2021.

#### Grants

For HAI grants, we employed the FoR system to define the dataset and retrieve data from Dimensions. We selected patents based on FoR, intersecting “11 Medical and Health Sciences” and “0801 Artificial Intelligence and Image Processing”. In this dataset, we aggregated the funding amounts (in USD) of HAI patents annually to visualize yearly trends of funding support in HAI. Ultimately, 6,113 HAI grants were indexed.

#### Clinical trials

We obtained HAI clinical trial data from Dimensions, which features data from over 671,000 clinical trials sourced from platforms such as International Standard Randomised Controlled Trial Number, ClinicalTrials.gov, EU Clinical Trials Register, Chinese Clinical Trial Registry, and others. Utilizing the FoR system, we identified 2,535 HAI clinical trials in Dimensions by intersecting “11 Medical and Health Sciences” and “0801 Artificial Intelligence and Image Processing”.

### Health indexer construction

Three major steps were followed in the process of health indexer construction: (a) Extracting (using natural language processing (NLP) tool to extract MeSH terms from abstracts and titles), (b) Mapping (mapping MeSH terms to 2-digit sub-categories), and (c) Integrating (connecting data from different sources and formats).

Publications retrieved from Medline are automatically MeSH-indexed, meaning they are labeled with multiple MeSH terms. For publications obtained from Dimensions but not indexed in Medline, we employed the NLM-developed NLP tool, MTI, to extract MeSH terms from titles and abstracts. Furthermore, all grants, patents, and 1,487 HAI clinical trials lacked recorded MeSH terms. In these cases, we also utilized the MTI tool to extract MeSH terms from the respective HAI documents.

MeSH descriptors are structured in a tree hierarchy. For each MeSH term, we stored its parent categories and sub-categories with the aim of connecting data across multiple levels and perspectives. For instance, under the MeSH hierarchy, all sub-categories of “Cardiovascular Diseases [C14]” are mapped to C14, ensuring that when retrieving cardiovascular-related data, all data under the MeSH term C14 are automatically queried. Additionally, we establish a mapping system among MeSH, SNOMED CT, and ICD-10 to minimize misunderstandings between different medical terminologies.

In this study, the term “integrating” refers to the methodology employed to index HAI documents by HAI subcategories (e.g., “Neurosciences”, “Cardiovascular Diseases”, “Machine Learning”, or “Robotics”). We utilized MeSH terms as the primary connectors for all data sources, enabling users to query HAI publications, patents, grants, and clinical trials using any combination of MeSH terms. Additionally, the dataset is indexable via ICD-10 and SNOMED CT.

### Defining health problems and AI technologies

Initially, we explored the FoR system provided by Dimensions to examine research trends. We employed the sub-levels of “11 Medical and Health Sciences” to represent health problems, which are oriented toward specialties rather than specific diseases. Table 1 displays the medical specialties under the FoR classification “11 Medical and Health Sciences”.

**Table 1.** Medical specialties in the FoR system

1101	Medical biochemistry and metabolomics
1102	Cardiorespiratory medicine and hematology
1103	Clinical sciences
1104	Complementary and alternative medicine
1105	Dentistry
1106	Human movement and sports science
1107	Immunology
1108	Medical microbiology
1109	Neurosciences
1110	Nursing
1111	Nutrition and dietetics
1112	Oncology and carcinogenesis
1113	Ophthalmology and optometry
1114	Pediatrics and reproductive medicine
1115	Pharmacology and pharmaceutical sciences
1116	Medical physiology
1117	Public health and health services
1199	Other medical and health sciences

However, due to the absence of sub-categories under “0801 Artificial Intelligence and Image Processing”, we were not able to classify documents by specific AI technologies using the FoR system. Consequently, we introduced the method of identifying AI technologies in HAI documents by utilizing the MeSH system.

As previously mentioned, the use of FoR to represent sub-categories of HAI documents does not adequately address the need for fine-grained classifications of both health problems and AI technologies. Therefore, with the assistance of the MeSH system, we enriched the annotations of HAI documents with disease-oriented classifications and more specific AI technology categories.

#### Health problems

MeSH descriptors are structured in a tree-like system, featuring 16 primary categories, including Organisms, Diseases, Drugs, Chemicals, etc. Given our HAI definition strategy, which focuses on broad health problems rather than specific diseases, we employed the 2-digit categories, such as “Nervous System Diseases [C10]”. Furthermore, for all sub-categories of the 2-digit categories, we mapped them to their respective parent 2-digit categories. Table 2 presents the complete categories for health problem classification.

**Table 2.** Health problems classification based on the MeSH system

Diseases	Infections [C01]
Neoplasms [C04]
Musculoskeletal diseases [C05]
Digestive system diseases [C06]
Stomatognathic diseases [C07]
Respiratory tract diseases [C08]
Otorhinolaryngologic diseases [C09]
Nervous system diseases [C10]
Eye diseases [C11]
Urogenital diseases [C12]
Cardiovascular diseases [C14]
Hemic and lymphatic diseases [C15]
Congenital, hereditary, and neonatal diseases and abnormalities [C16]
Skin and connective tissue diseases [C17]
Nutritional and metabolic diseases [C18]
Endocrine system diseases [C19]
Immune system diseases [C20]
Disorders of environmental origin [C21]
Animal diseases [C22]
Pathological conditions, signs and symptoms [C23]
Occupational diseases [C24]
Chemically induced disorders [C25]
Wounds and injuries [C26]
Mental disorders	Mental disorders [F03]
Health care	Population characteristics [N01]
Health care facilities, manpower, and services [N02]
Health care economics and organizations [N03]
Health services administration [N04]
Health care quality, access, and evaluation [N05]
Environment and public health [N06]

#### AI technologies

Five types of health-related AI technologies were introduced by an existing research: expert systems, machine learning, natural language processing, automated planning and scheduling, and image signal processing [15]. In this study, based on the defining methodology, we came up with 5 new AI categories: decisive rules, knowledge bases, machine learning (deep learning), natural language processing, and robotics. Beyond a sole proposal, we pragmatically annotated most of the data with our classifications. The dataset also used MeSH descriptors to classify AI technologies in HAI publications. Compared to the strategy that uses literal MeSH categories for health problem classifications, we proposed 5 categories of AI technologies by grouping MeSH descriptors. In the MeSH tree structure, AI is located under the category Information Science, coded as L01.224.050.375, with its MeSH hierarchy as follows:

-Artificial Intelligence [L01.224.050.375]

-Computer Heuristics [L01.224.050.375.095]

-Expert Systems [L01.224.050.375.190]

-Fuzzy Logic [L01.224.050.375.250]

-Knowledge Bases [L01.224.050.375.480]

-Machine Learning [L01.224.050.375.530]

-Natural Language Processing [L01.224.050.375.580]

-Neural Networks, Computer [L01.224.050.375.605]

-Robotics [L01.224.050.375.630]

Drawing upon HAI applications and guidance from AI experts, we manually reorganized the sub-categories of AI MeSH descriptors into the categories presented in Table 3. It is important to note that these categories are not mutually exclusive; rather, they offer an overview of AI technology usage in the healthcare domain.

**Table 3.** AI technologies classification based on the MeSH system

Categories	MeSH descriptors
1	Decisive rules	Fuzzy logic [L01.224.050.375.250]
Computer heuristics [L01.224.050.375.095]	
Expert systems [L01.224.050.375.190]	
2	Knowledge bases	Knowledge bases [L01.224.050.375.480]
Biological ontologies [L01.224.050.375.480.500]	
Gene ontology [L01.224.050.375.480.500.500]	
3	Machine learning (deep learning)	Machine learning [L01.224.050.375.530]
Deep learning [L01.224.050.375.530.250]	
Supervised machine learning [L01.224.050.375.530.500]	
Support vector machine [L01.224.050.375.530.500.500]	
Unsupervised machine learning [L01.224.050.375.530.750]	
Neural networks, computer [L01.224.050.375.605]	
Deep learning [L01.224.050.375.605.500]	
4	Natural language processing	Natural language processing [L01.224.050.375.580]
5	Robotics	Robotics [L01.224.050.375.630]

By leveraging the PubMed website and the MTI tool, we successfully annotated HAI documents with 100% coverage of MeSH terms and a substantial proportion of HAI categories (75.12% on average, 92.9% for publications). Table 4 illustrates the specific extraction and mapping rates for each HAI document type.

**Table 4.** MeSH terms and HAI categories coverage. Total: total number of the document type. Number of null: the number of documents without a MeSH term. MTI extracted: the number of extracted MeSH terms from the nulls using MTI. HAI mapped: total number of publications with an HAI sub-category. Total mapping rate: $\frac{\mathrm{HAI}\;\text{mapped}}{\text{Total}}.$

	MeSH terms extracting and mapping statistics					
	Total	Number of null	MTI extracted	MeSH labeled	HAI mapped	Total mapping rate
Publication	75,820	23,108	23,108	75,820	70,401	92.9%
Patent	11,226	11,226	11,226	11,226	6,001	53.5%
Grant	6,113	6,113	6,113	6,113	4,514	73.8%
Clinical trials	2,535	1,487	1,487	2,535	2,139	84.4%
Dataset	638	638	638	638	453	71.0%

Different medical terminologies convey different meanings in complexity and specificity [16]. To make up the understanding gaps among the terminologies, we enriched the HAI dataset with mapped health terminologies including SNOMED CT and ICD-10 using the UMLS API [17]. Table 5 shows the coverage of each terminology.

**Table 5.** Mapping rate of HAI documents using different terminologies

	MeSH	ICD-10	SNOMED CT
Publication	100%	74.29%	96.93%
Patent	100%	76.40%	95.24%
Grant	100%	83.69%	99.54%
Clinical trial	100%	87.90%	97.71%
Dataset	100%	78.68%	92.95%

## Results

### Data description

#### Fields of data

In this section, we introduce the field names, applicable documents, types, and descriptions of the HAI dataset, which encompasses 62 fields and 10 data types. To ensure transparency and accessibility for other researchers, we present detailed information in Table 6. Applicable documents are denoted as P1 (publication), D (dataset), P2 (patent), G (grant), and C (clinical trial). Aside from the fields unique to this study (health_terms_name, mapped_ai_terms, mapped_mesh_terms, m_aff_country, ICD-10, and snomed_ct), the remaining fields, along with their corresponding types and descriptions, are sourced from Dimensions.ai. A more comprehensive introduction to the fields can be found at https://docs.dimensions.ai/dsl/data-sources.html.

**Table 6.** Fields and descriptions

Field_name	Document	Type	Description
category_bra	P1, D, P2, G, C	Categories	Broad research areas
category_for	P1, D, P2, G, C	Categories	ANZSRC fields of research classification
category_hra	P1, D, P2, G, C	Categories	Health research areas
category_hrcs_hc	P1, D, P2, G, C	Categories	HRCS—health categories
category_hrcs_rac	P1, D, P2, G, C	Categories	HRCS—research activity codes
category_icrp_cso	P1, D, P2, G, C	Categories	CRP common scientific outline
category_icrp_ct	P1, D, P2, G, C	Categories	ICRP cancer types
category_rcdc	P1, D, P2, G, C	Categories	Research, condition, and disease categorization
category_sdg	P1, D, P2, G, C	Categories	SDG—sustainable development goals
funder_countries	P1, D, P2, G, C	Countries	The country of the funding organization
funders	P1, D, P2, G, C	Organizations	GRID organizations
health_terms_name	P1, D, P2, G, C	String	The mapped health problems
id	P1, D, P2, G, C	String	Document ID
m_aff_country	P1, D, P2, G, C	String	Cleaned and curated affiliated country
mapped_ai_terms	P1, D, P2, G, C	String	Mapped AI technologies of the document
mapped_health_terms	P1, D, P2, G, C	String	Mapped health problems of the document
*mapped_mesh_terms*	P1, D, P2, G, C	String	Parents of the mesh terms
*mesh_terms*	P1, D, P2, G, C	String	The mesh terms of the document
*ICD-10*	P1, D, P2, G, C	Json	Mapped ICD-10 codes
*snomed_ct*	P1, D, P2, G, C	Json	Mapped SNOMED CT concepts
researchers	P1, D, P2, G, C	Researchers	Researchers IDs associated to the document
title	P1, D, P2, G, C	String	The title of the document
abstract	P1, P2, G, C	String	Abstract of the document
date	P1, D, P2	Date	publication/filing date
year	P1, D, P2	Integer	The year the document was published/filed
linkout	P1, G, C	String	Original URL for the document
research_orgs	P1, D, C	Organizations	GRID organizations
times_cited	P1, P2	Integer	The number of times the document has been cited
category_uoa	P1, G	Categories	Units of assessment
authors	P1, D	Json	Ordered list of the dataset authors.
doi	P1, D	String	Digital object identifier
journal	P1, D	String	The journal a publication/dataset belongs to
associated_grant_ids	P1, D, C	String	IDs of the grants associated to the document
assignee_names	P2	String	Name of assignees of the patent
assignees	P2	Organizations	Disambiguated GRID organizations who own or have owned the rights of a patent
cpc	P2	String	Cooperative patent classification categorization
expiration_date	P2	Date	Date when the patent expires
filing_status	P2	String	Filing status of the patent
granted_year	P2	Integer	The year on which the official body grants the patent
inventor_names	P2	String	Names of the people who invented the patent
ipcr	P2	String	International patent classification reform categorization
legal_status	P2	String	The legal status of the patent
publication_date	P2	Date	Date of publication of a patent
altmetric	P1	Float	Altmetric Attention Score
pmcid	P1	String	PubMed Central ID
pmid	P1	String	PubMed ID
relative_citation_ratio	P1	Float	Relative citation performance of article when compared to similarly aged articles in its area of research
type	P1	String	Publication type
end_date	G	Date	Date when the grant ends
start_date	G	Date	Date when the grant starts
start_year	G	Integer	Year when the grant starts
associated_publication_id	D, P2, C	String	ID of the publication linked to the document
description	D	String	Description of the dataset
figshare_url	D	String	Figshare URL for the dataset
keywords	D	String	Keywords used to describe the dataset (from authors)
license_name	D	String	The dataset license name
license_url	D	String	The dataset license URL
repository	D	Repositories	Repository associated with the dataset
active_years	C	Integer	List of active years for a clinical trial
conditions	C	String	List of medical conditions names
gender	C	String	The gender of the clinical trial subjects
investigators	C	Json	Additional details about investigators, including affiliations and roles
phase	C	String	Phase of the clinical trial, as a string
registry	C	String	The platform where the clinical trial has been registered

#### Document interconnections

**Fig. 3.**
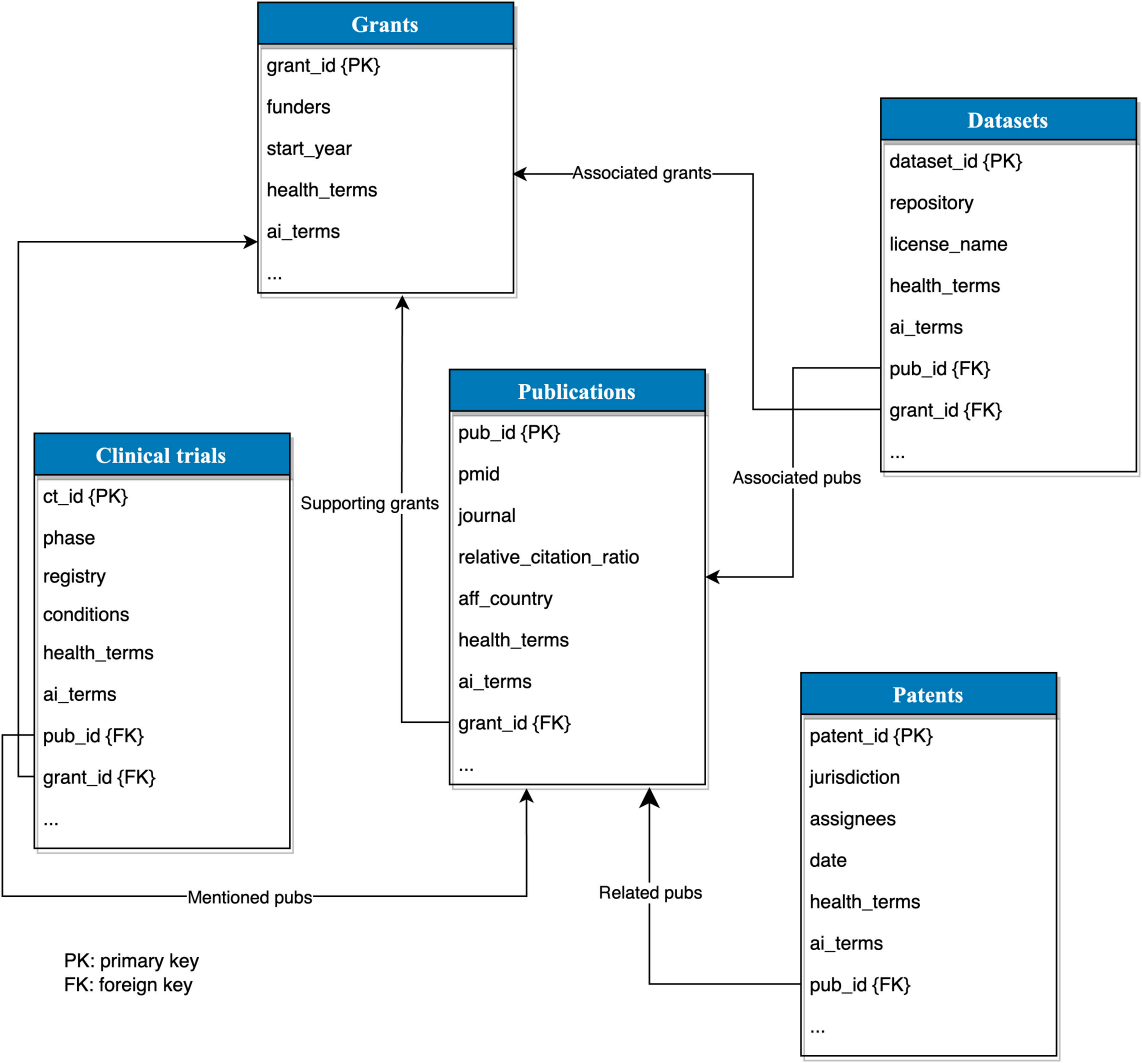
Relationship diagram of HAI documents. PK, primary key; FK, foreign key.

Figure 3 presents a comprehensive diagram showcasing the intricate relationships between various HAI documents. The visual representation effectively highlights the interconnected nature of the HAI field, demonstrating how different documents contribute to and build upon one another to advance our understanding of AI applications in healthcare. The diagram enables researchers, practitioners, and other stakeholders to better grasp the complex landscape of HAI literature, identify key themes and trends, and, ultimately, streamline their search for relevant information. By exhibiting the relationships between these documents, this study serves as a valuable tool for fostering cross-disciplinary collaboration and facilitating the development of innovative solutions in the rapidly evolving field of health AI.

#### HAI landscape: Heatmap analysis

**Fig. 4.**
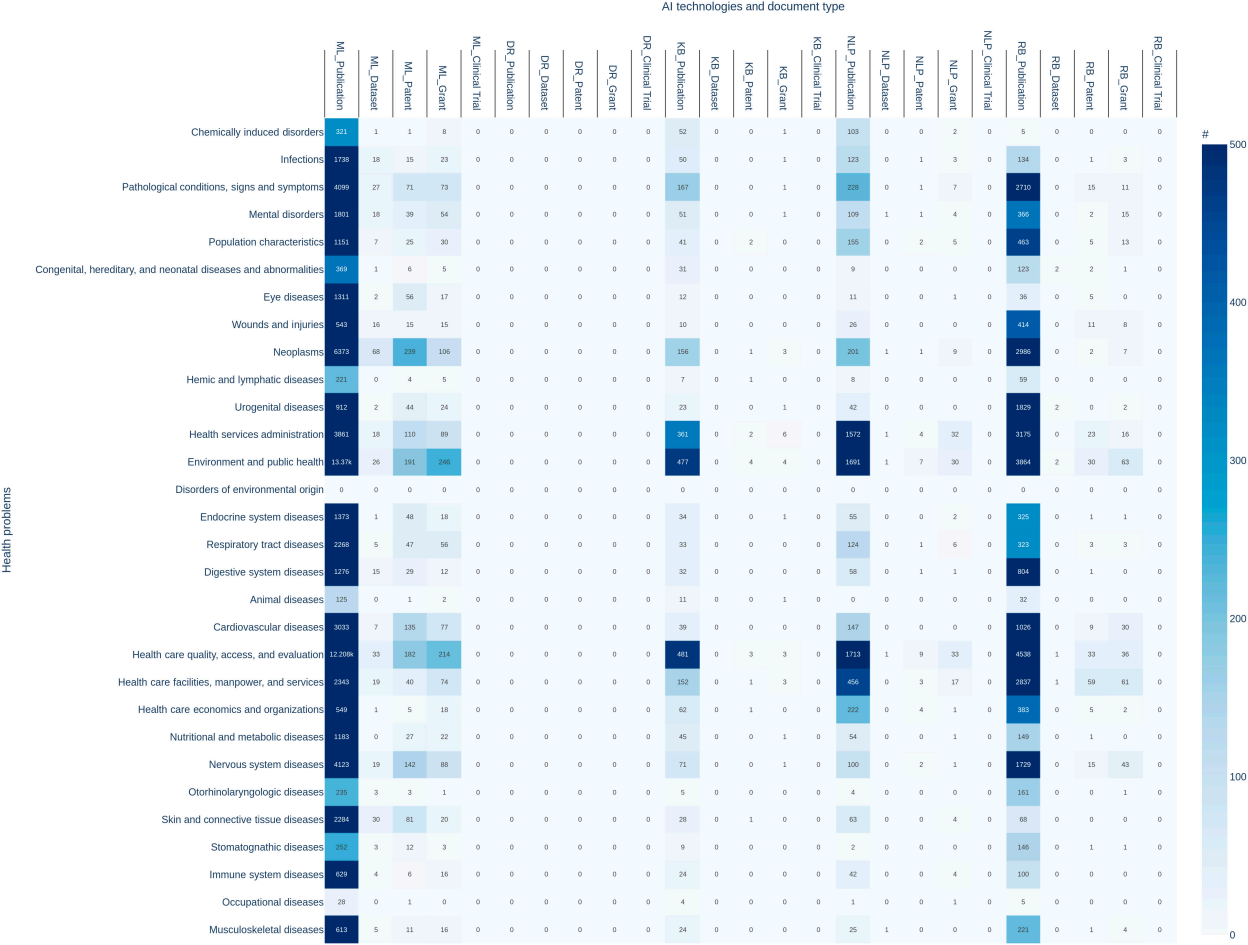
Evidence heatmap based on all HAI documents.

In addition, we have developed an HAI evidence heatmap (Fig. 4) to identify the most prevalent areas and provide researchers with an overview of under-explored topics. The *x*-axis displays various AI technologies (ML: machine learning, DR: decision rule, KB: knowledge base, NLP: natural language processing, and RB: robotics) alongside the document types (publication, dataset, patent, grant, and clinical trial). The *y*-axis represents the health issues being addressed. Each cell in the heatmap contains a number indicating the total count of publications for a specific combination. The extent of the color signifies the volume of documents, with the deepest blue indicating a count of 500 or more.

### Data analysis

#### Worldwide trend

**Fig. 5.**
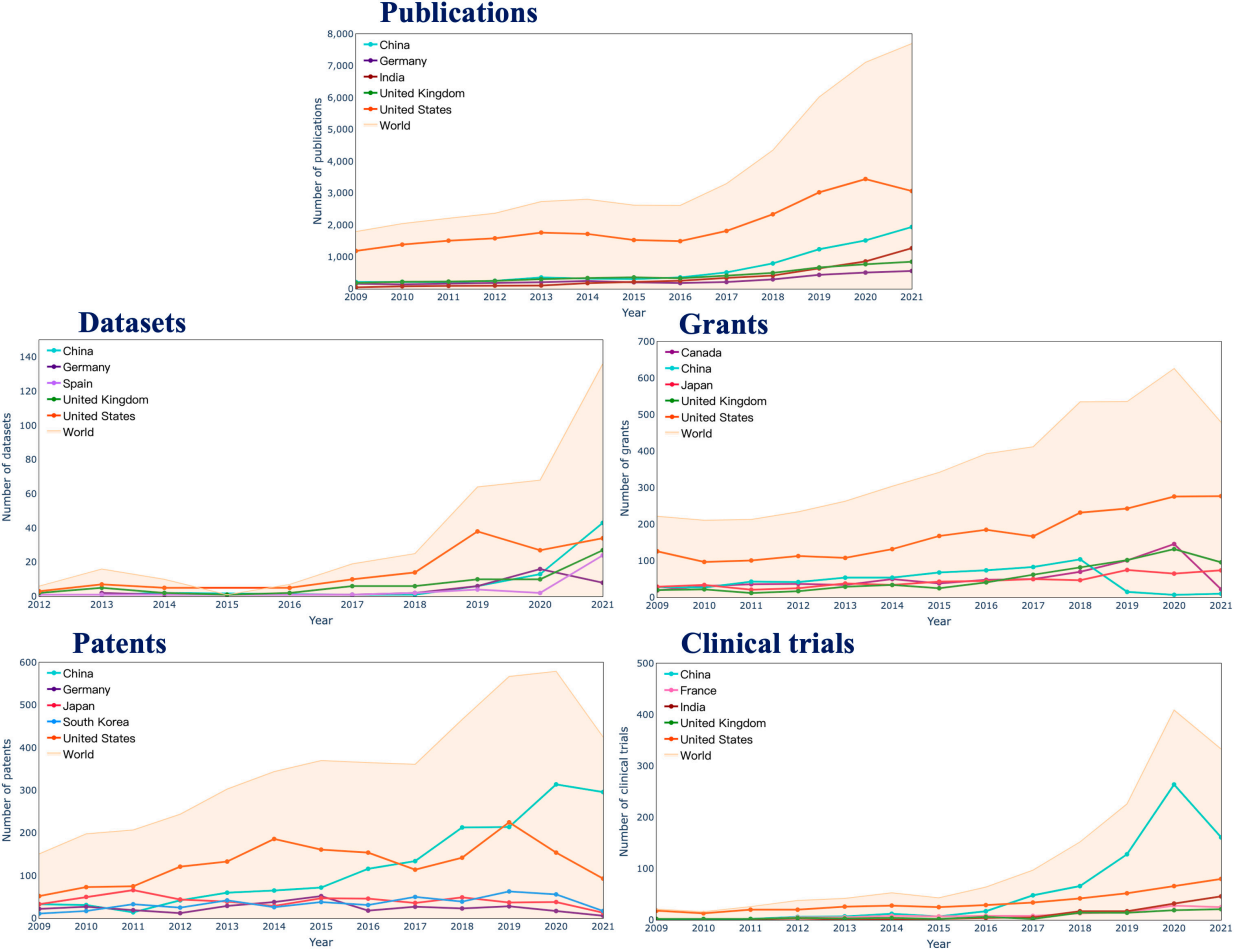
Worldwide trend of the numbers of HAI documents.

In Fig. 5, we present a visualization of the global trends in the number of HAI documents from 2009 to 2021. Regarding publications, the United States has consistently maintained its leading position, followed by China, while India experienced considerable growth in 2021. With respect to open research datasets, the United States initially witnessed a surge in 2019, only to be surpassed by China in 2021. In terms of grants, the United States has consistently held the top position, similar to its performance in publications. China's grant numbers, however, declined significantly after 2018, with Japan, the United Kingdom, and Canada demonstrating comparable figures. Pertaining to patents, the United States and China have alternated as leaders, with China ultimately securing the lead in 2021, significantly outpacing other countries. Lastly, in HAI clinical trials, China overtook the United States in 2017, maintaining its leading position despite a marked decrease in 2021. The United States ranked second, followed by India, France, and the United Kingdom.

#### Open research datasets and clinical trials

**Fig. 6.**
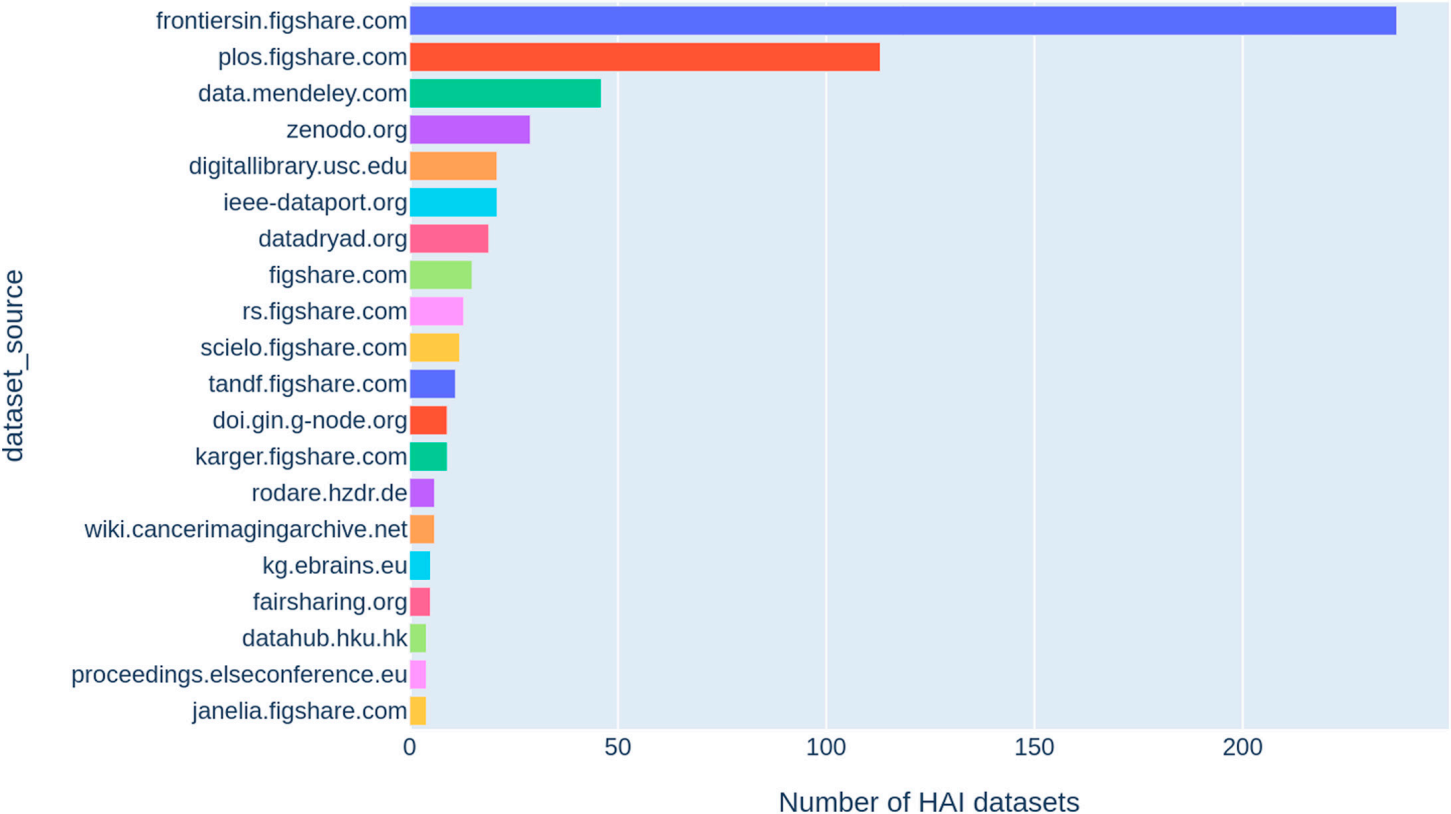
The distribution of repositories of HAI open research datasets.

To optimize utility for researchers, we aggregated HAI documents into concise dataframes. An example dataframe of datasets derived from the curated HAI dataset is presented in Table 7. This enables researchers to efficiently access datasets and repositories relevant to particular health issues or AI technologies. Furthermore, we examined the distribution of HAI dataset sources, illustrating the most prominent repositories contributing to the HAI research domain (Fig. 6). The figure reveals that Frontiers (https://frontiersin.figshare.com/) serves as the foremost repository for HAI dataset publication, succeeded by PLOS (https://plos.figshare.com/) and Mendeley (https://data.mendeley.com).

**Table 7.** Example HAI research datasets

Dataset_id	Title	Source_repo	Health_problems	AI technologies
dataset.8683855	Neural network cascade optimizes MicroRNA Biomarker selection for nasopharyngeal cancer prognosis	plos.figshare.com	[“Neoplasms”, “otorhinolaryngologic diseases”, “stomatognathic diseases”]	[“Machine learning (includes deep learning)”]
dataset.7557580	Table_3_Application of neural network and cluster analyses to differentiate TCM patterns in patients with breast cancer.docx	frontiersin.figshare.com	[“Neoplasms”, “health care quality, access, and evaluation”, “skin and connective tissue diseases”, “environment and public health”]	[“Machine learning (includes deep learning)”]
dataset.25958850	Data_Sheet_1_Using machine learning algorithms to predict Candidaemia in ICU patients with new-onset systemic inflammatory response syndrome.docx	frontiersin.figshare.com	[“Health care facilities, manpower, and services”, “pathological conditions, signs and symptoms”, “infections”]	[“Machine learning (includes deep learning)”]
dataset.57695649	Table_6_Value of the application of CE-MRI radiomics and machine learning in preoperative prediction of sentinel lymph node metastasis in breast cancer.xlsx	frontiersin.figshare.com	[“Neoplasms”, “pathological conditions, signs and symptoms”, “skin and connective tissue diseases”]	[“Machine learning (includes deep learning)”]
dataset.25720939	Data_Sheet_1_Prediction of tumor shrinkage pattern to neoadjuvant chemotherapy using a multiparametric MRI-based machine learning model in patients with breast cancer.pdf	frontiersin.figshare.com	[“Neoplasms”, “skin and connective tissue diseases”]	[“Machine learning (includes deep learning)”]

AI has been employed in various aspects of clinical trials, such as recruitment, design, data analysis, and drug development, among others. As a prominent digital health technology (DHT), AI necessitates systematic evaluation within clinical environments. In this section, we undertake a preliminary exploratory data analysis of clinical trials incorporating AI applications from diverse perspectives. Our objective is to stimulate further in-depth and systematic investigations in this field.

**Fig. 7.**
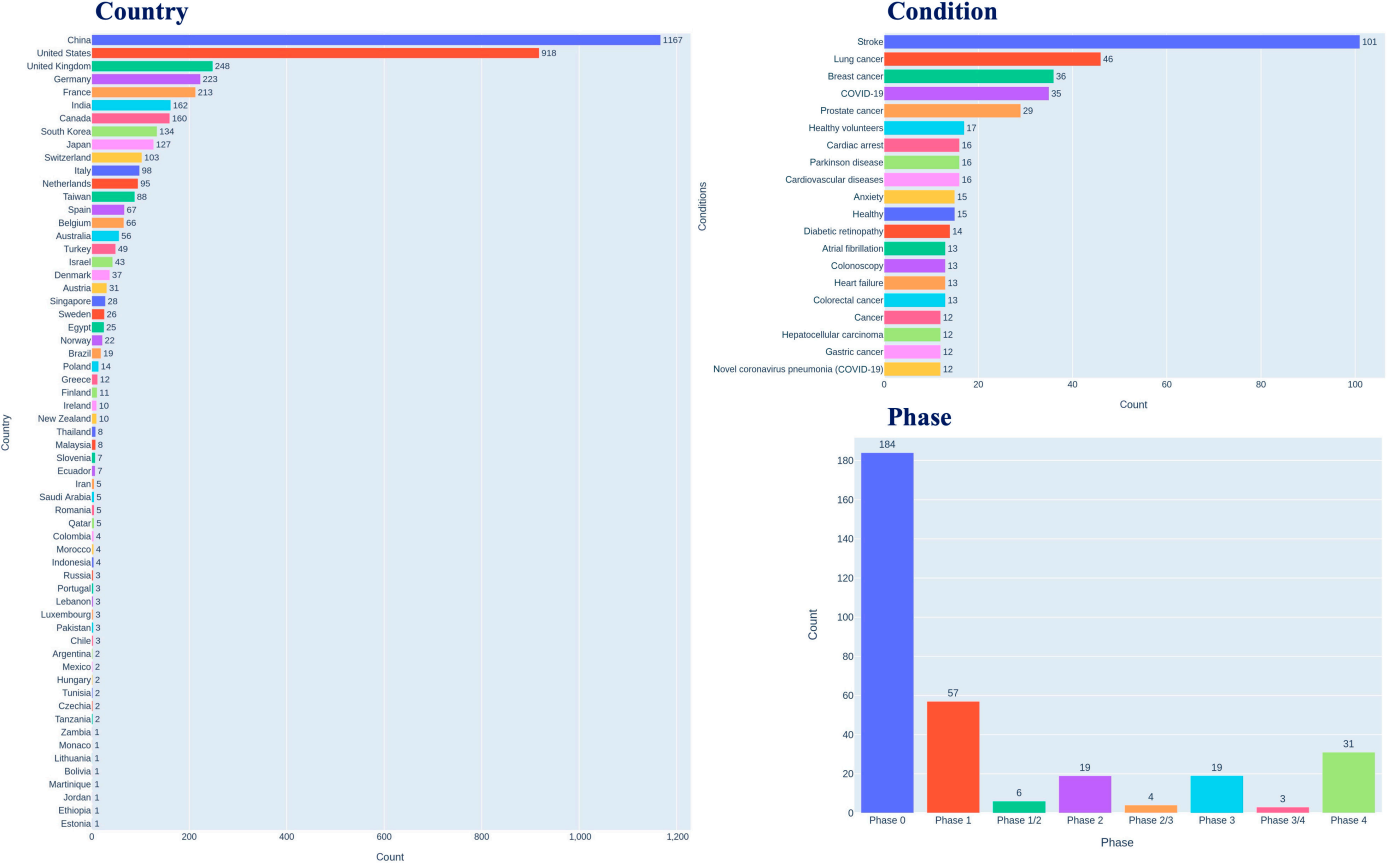
An overview of HAI clinical trials by country, condition, and phase.

As observed in Fig. 7, a substantial portion of AI clinical trials remain in the preliminary stages (Phase 0 and Phase 1), potentially encompassing activities such as dataset collection, machine learning model development, and initial data acquisition regarding AI performance. Simultaneously, a notable fraction of AI clinical trials has advanced to Phase 4, assessing the efficacy and safety of AI in relation to clinical outcomes. AI technology has been implemented in a diverse array of clinical conditions, with the top 5 being stroke, lung cancer, breast cancer, COVID-19, and prostate cancer. Among the research organizations' countries involved in AI clinical trials, China and the United States stand out as frontrunners in terms of the number of AI clinical trials conducted.

### Sankey: Health problems vs. AI technologies

**Fig. 8.**
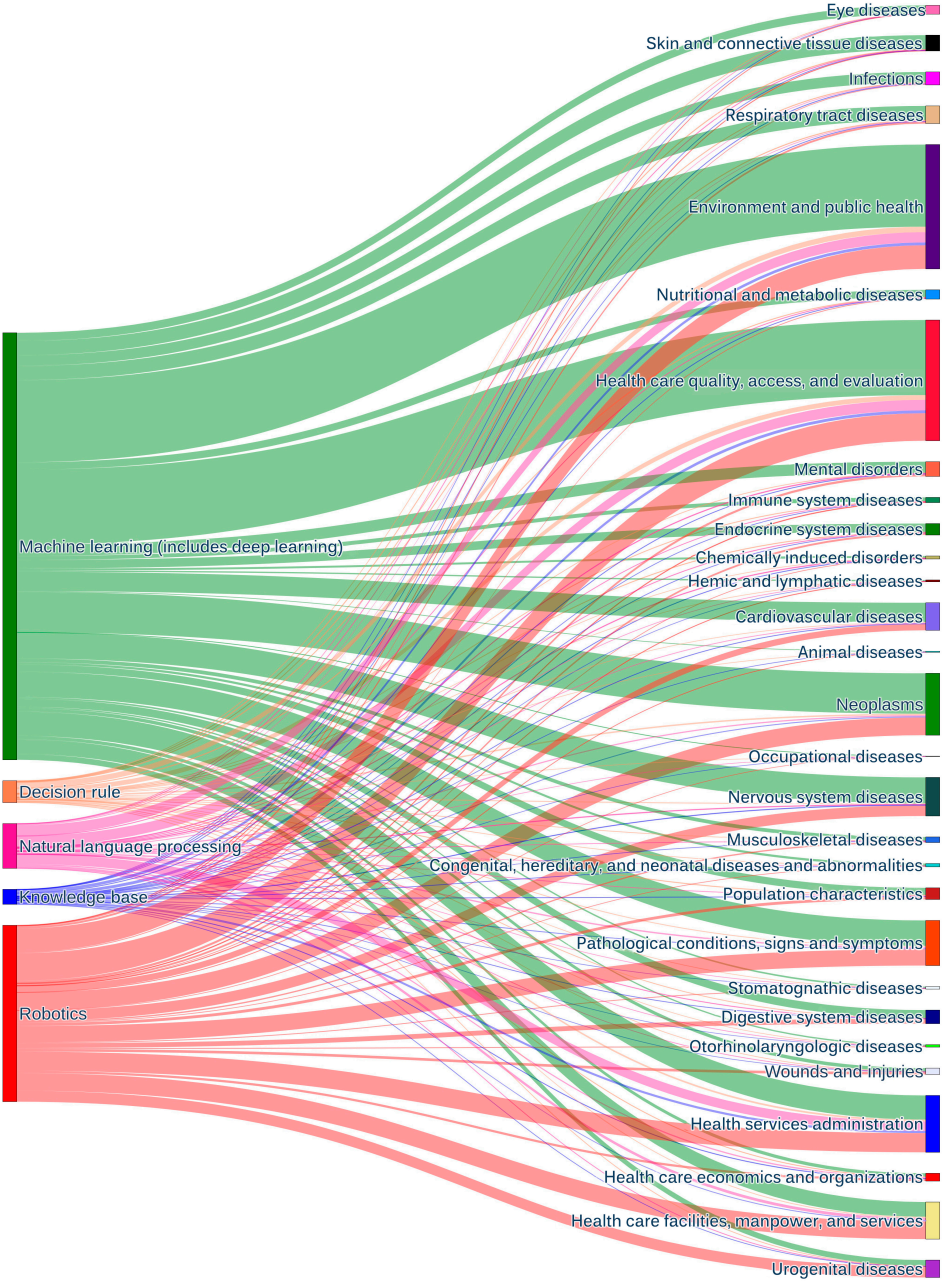
Sankey diagram of all HAI documents. Left: AI technologies. Right: Health problems.

The utilization of AI technologies across distinct health issues can be examined thoroughly by investigating flows between sub-categories. In this study, we constructed a Sankey diagram (Fig. 8) based on the co-occurrence of HAI sub-categories, offering a comprehensive view of the translational relationships between health problems and AI technologies in all HAI documents. Machine learning emerges as the principal AI technology employed across the majority of health issues, with robotics also playing a significant role in areas such as “Health Care Facilities, Man Power, and Services” and “Neoplasms”. In contrast, knowledge bases constitute a relatively minor portion of AI technologies but maintain a presence in most health problems.

## Technical Validation

### Transparency and reproducibility

We used data from well-known public sources like PubMed and Dimensions. This ensures that anyone can access and verify the data. The dataset employs standard medical vocabularies like MeSH and FoR, aligning it with common biomedical language. This makes the data more understandable and useful in the field. Additionally, we processed the data using the MTI, a widely used tool in biomedical research. This choice ensures that our methods align with established practices, making the dataset more credible and easier for other researchers to replicate and validate in their studies.

### Limitation

We have to point out the limitations and biases in this dataset. (a) The coverage of MeSH terms in PubMed is limited to Medline publications. Thus, documents without a MeSH term were potentially not detected during data acquisition. (b) The extraction of MeSH terms in Medline publications is currently machine-indexed, causing the lacking of accuracy and coverage (0.61 in precision and 0.56 in recall, respectively)[18]. Compared to human indexing, MTI tends to produce more specific terms, which was inconsistent to specificity principles [19]. (c) The FoR classification system used in Dimensions is machine learning algorithm based, displaying a potential inherent bias produced in the data curation [20,21].

Despite the limitations of dataset curation in this study, we utilized the most comprehensive data platforms and widely used tools to ensure the standability, transparency, and reproducibility. Also, the automatic pipeline to build the dataset guarantees the efficiency for refinements and improvements in future research.

## Summary

In this study, we acquired HAI documents by designing a definition strategy with data from PubMed and Dimensions. Compared to qualitative analysis of AI trends in health care, we proposed a pipeline to construct a dataset with all types of related documents including publications, datasets, patents, grants, and clinical trials from different scholarly resources. Besides, with a curated sub-category classification, this dataset supports indexing the HAI and retrieving all the related information such as health problems, ICD code, AI technology, etc. We observed and discovered evidence structures in HAI documents including datasets and clinical trials, hopefully giving intuitions of how researchers can take advantage of this dataset, providing researchers in the areas of both health and computer science an efficient tool for information collection, bibliographic analysis, and knowledge discovery in HAI.

### Ethical declarations

In this research, we utilized publicly available data, including publications and clinical trials, which do not contain sensitive personal health information. Therefore, traditional concerns about data privacy and informed consent are not directly applicable.
